# Autologous stem cell transplantation for pediatric solid tumors in a resource-limited setting: a single-center experience of 15 years

**DOI:** 10.3389/fonc.2026.1796956

**Published:** 2026-06-16

**Authors:** Debabrata Mohapatra, Deepam Pushpam, Sameer Bakhshi, Ranjit Kumar Sahoo, Surender K. Sharawat, Sandeep Agarwala

**Affiliations:** 1 Department of Medical Oncology, Dr. B.R. Ambedkar Institute Rotary Cancer Hospital (IRCH), All India Institute of Medical Sciences (AIIMS), New Delhi, India; 2 Department of Pediatric Surgery, All India Institute of Medical Sciences (AIIMS), New Delhi, India

**Keywords:** autologous stem cell transplant, Ewing sarcoma, germ cell tumors, low- and middle-income countries, neuroblastoma, pediatric solid tumors

## Abstract

**Introduction:**

Autologous hematopoietic stem-cell transplant(ASCT) following high-dose chemotherapy is an established treatment for selected pediatric solid tumors. However, data from low- and middle-income countries(LMICs) remain scarce, where outcomes may be influenced by limited supportive care infrastructure and higher infection risks. Most available reports focus primarily on neuroblastoma, with little evidence for other solid tumors.

**Methods:**

The study included children, adolescents, and young adults (CAYA,0–25 years) with solid tumors treated at a tertiary-care center in India, aiming to assess survival outcomes, engraftment kinetics, and toxicities. Data were collected from patient files and electronic databases for those who underwent ASCT between January 2008 and June 2025.

**Results:**

Out of 92 patients, median age was 6y (IQR,4-16) with 69.6% males. Diagnoses included: high-risk neuroblastoma(HRNB,52.2%), relapsed/refractory Ewing sarcoma(EWS,22.8%), relapsed/refractory germ-cell tumors(GCT,15.2%), retinoblastoma(RB,5.4%), and soft-tissue sarcoma (STS,4.4%). Forty-three (46.8%) underwent ASCT in relapse/refractory setting, while 49(53.2%) had upfront ASCT. Median CD34+cell dose was 5.7(IQR,3.5-6.9) million/kg, with a median cryopreservation duration of 16(IQR,8-49) days. Median time to neutrophil-engraftment was 11(IQR,9-12) days. The 3-year OS of the entire cohort was 50.6% (95%CI: 36.8-62.9), and EFS was 35.9% (95%CI: 23.6-48.3). Three-year OS for HRNB, relapsed/refractory-EWS, relapsed/refractory-GCT, STS and RB were 48.5%(95%CI: 29.2-65.3), 43.8% (95%CI: 17.8-67.4), 56.3%(95%CI: 20.9-80.9), 66.7% (95%CI: 5.4-94.5), and 50.0%(95%CI: 6.0-91.0), respectively. For HRNB and EWS, BuMel-140 had better OS than CEM-200 [HR = 0.15,95%CI:0.04-0.7,p=0.005].

**Conclusion:**

ASCT yields acceptable outcomes for selected high-risk or relapsed/refractory pediatric solid tumors in LMICs, with outcomes in GCT comparable to global standards, based on experience from a high-volume transplant center.

## Introduction

The five-year survival of pediatric cancers has exceeded 80% in developed countries due to improved systemic therapy and supportive care in recent times, although outcomes in the low- and middle-income countries (LMICs), including India, are still lagging (1, 2). In contrast, the outcomes of some high-risk pediatric solid tumors like high-risk neuroblastoma (HRNB), relapsed/refractory sarcomas, and germ-cell tumors (GCT), etc., remain suboptimal due to aggressive biology, high relapse rates, and resistance to conventional doses of chemotherapy. One potential strategy to overcome tumor resistance to conventional-dose chemotherapy involves the administration of myeloablative high-dose chemotherapy (HDC) followed by hematopoietic rescue using autologous hematopoietic stem cells, which can be collected either from bone marrow or from peripheral blood after appropriate mobilization. This procedure is termed autologous hematopoietic stem cell transplantation (ASCT). ASCT has been employed in several pediatric solid tumors, with randomized trials showing improved outcomes in high-risk neuroblastoma and high-risk localized Ewing sarcoma, while a similar trial is ongoing for relapsed GCT (3–5).

However, all these data originated from high-income countries (HICs), and there remains a significant gap in LMICs, including India, where the childhood cancer burden is high but access to transplant is limited. The available reports from LMICs are limited, often focusing mainly on neuroblastoma, with limited outcome data in Ewing sarcoma, only isolated case reports in relapsed/refractory GCTs, and virtually no systematic evidence in soft-tissue sarcomas and retinoblastoma. Transplant-related mortality in LMICs may be higher due to infection-related complications, compounded by limited access to HEPA-filtered transplant units. The available reports from LMICs often do not capture engraftment kinetics, transplant-related toxicity, or long-term outcomes, which may differ from HICs due to variable supportive care and infrastructure.

In view of the paucity of data from LMICs on ASCT in solid tumors, this study was designed to evaluate real-world survival outcomes and toxicity profile of ASCT in pediatric solid tumors in resource-constrained setting, analyze the impact of conditioning regimens and compare results with the published literature.

## Methods

### Design and ethical considerations

It was a retrospective study and was conducted in compliance with the Declaration of Helsinki. Prior Institutional Review Board approval was obtained (*AIIMSA2075/26.09.2024*).

### Study population

The study included children, adolescents, and young adults (CAYA) aged ≤25 years who underwent ASCT for solid tumors, including high-risk neuroblastoma, relapsed/refractory GCTs, Ewing sarcoma, other soft-tissue sarcomas, and retinoblastoma between January 2008 and June 2025. Given the limited availability of large-scale outcome data on ASCT for pediatric solid tumors from LMICs, and the relative rarity of transplant indications across certain individual tumor subtypes, a diagnosis-inclusive cohort was analyzed to reflect real-world practice rather than disease-specific efficacy.

### Conditioning regimens

After induction chemotherapy and response assessment, patients with adequate performance status and acceptable cardiac, renal, hepatic, and pulmonary function were selected for ASCT based on disease-specific risk stratification and chemosensitivity. HRNB patients underwent ASCT after achieving at least a partial response to induction therapy, with clearance of bone marrow disease, while ASCT in relapsed or refractory Ewing sarcoma and GCT was limited to those with chemosensitive disease following salvage therapy.

Patients underwent peripheral blood stem cell harvest on an outpatient basis following G-CSF mobilization, with selective use of plerixafor for anticipated or documented poor mobilization, introduced from January 2016. Given the constraints of limited transplant beds, stem cells were routinely cryopreserved, and patients were subsequently admitted to the transplant unit for ASCT at the next available slot. For neuroblastoma and Ewing sarcoma, melphalan at a dose of 200mg/m^2^ along with carboplatin and etoposide (CEM-200) was used for conditioning till November 2011. Later, it was changed to melphalan at a dose of 140mg/m^2^ along with busulfan at a dose of 16mg/kg (BuMel-140), after the initial reports of superiority of BuMel-140, by Ladenstein et al. (6). For relapsed/refractory GCTs and retinoblastoma, carboplatin and etoposide-based conditioning regimens were used. Detailed conditioning regimens are provided in **Supplementary Table 1**.

### Supportive care

Supportive care included premptive transfusions of platelets and packed red blood cells, as well as prophylactic antimicrobials, such as antifungals (e.g., fluconazole) and antivirals (e.g., acyclovir), along with post-transplant G-CSF until neutrophil engraftment. Additionally, cotrimoxazole prophylaxis for *Pneumocystis carinii* was administered for 3 months after engraftment. Routine antibacterial prophylaxis was not administered because of concerns regarding antimicrobial resistance and institutional preference for early empiric therapy based on clinical assessment.

Chlorhexidine mouthwash and sitz baths were administered at least twice daily. While enteral nutrition was the preferred route, parenteral nutrition was initiated liberally in patients with compromised enteral intake lasting more than 72 hours. The facility did not have HEPA filtration. To minimize the risk of infections, stringent aseptic measures and hand hygiene protocols were consistently enforced.

### Logistics and cost of ASCT

The estimated cost of every ASCT procedure was approximately ₹3,00,000 (~3,500 USD). This included hospitalization, chemotherapy, stem cell harvest, cryopreservation, and supportive care. Importantly, out-of-pocket expenses were negligible because most of the patients were supported by government funding mechanisms such as the Health Minister’s Discretionary Grant (HMDG), state-level health assistance programs, and the national *Ayushman Bharat–Pradhan Mantri Jan Arogya Yojana* (AB-PMJAY) public insurance scheme. Also, several non-governmental organizations (NGOs) contributed additional costs, which facilitated access to ASCT for families from diverse socioeconomic backgrounds.

### Definitions

Engraftment was defined as the first of three consecutive days on which the absolute neutrophil count (ANC) is >500/microliter for granulocytes and on which the platelet count is >20,000/microliter without transfusion dependence, for platelets (7).

### Data collection and statistical-analysis

A retrospective chart review was conducted using manual and electronic hospital records. Data collection included baseline demographics, diagnosis, pre-ASCT remission status, CD34+cell dose, conditioning regimen, and complications. Statistical analysis was done using Stata version 12.0 (StataCorp, College Station, TX, USA).

Descriptive statistics included median values for continuous variables and proportions for categorical variables. Event-free survival (EFS) and overall survival (OS) were calculated from the day of ASCT using the Kaplan-Meier survival analysis. For EFS, any death, relapse, or progression was considered an event. Outcomes were censored on the 31^st^ August 2025. OS and EFS were calculated for the entire cohort and also disease-wise. To explore predictive variables for survival, univariate and multivariate Cox proportional hazards regression analyses were performed, including baseline characteristics, remission status, and conditioning regimen. Toxicity data included infectious and non-infectious complications, and transplant-related mortality (TRM).

## Results

### Baseline patient characteristics

A total of 92 children, adolescents, and young adults (CAYA) underwent ASCT for solid tumors over the above-mentioned study period. The median age at transplant was 6 years (IQR, 4–16 years), with the majority being male (69.6%). High-risk neuroblastoma (HRNB) was the most common indication of ASCT, accounting for 48 patients (52.2%), followed by relapsed/refractory Ewing sarcoma (EWS) in 21 (22.8%), germ cell tumors (GCT) in 14 (15.2%), retinoblastoma (RB) in 5 (5.4%), and soft-tissue sarcoma (STS) in 4 patients (4.4%). Baseline characteristics, including diagnosis, treatment setting (first-line vs relapsed/refractory), and remission status at ASCT, are summarized in **Table 1**.

**Table 1:** Baseline characteristics and toxicity details of the patient cohort.

Variable	Number (N = 92)	Percentage (%)
Age		
Median (IQR)	6 yrs. (4-16)	
Sex		
Male	64	69.6
Diagnosis		
Neuroblastoma	48	52.2
Ewing sarcoma	21	22.8
Germ cell tumor	14	15.2
Retinoblastoma	5	5.4
Soft-tissue sarcoma*	4	4.4
Indication and timing of transplant		
First remission	49	53.2
Relapse or refractory (post salvage)	43	46.8
Pre-ASCT disease status		
Complete remission (CR)	36	39.1
Not in complete remission (No CR)^#^	56	60.9
Cell dose and engraftment	Median (IQR)	Range
Median CD34 in 10^6^/kg	5.7 (3.5-6.9)	1.8-20.1
Median duration of cryopreservation in days	16 (8-49)	3-169
Median hospitalization duration in days	23 (20-28)	12-41
Median time to ANC engraftment in days	11 (9-12)	8-48
Median number of PRBC transfusions needed	1 (1-2)	0-6
Median number of platelets (SDP) needed	2 (1-4.5)	1-15
Median duration of antibiotic days	10 (7-13)	4-42
Median duration of follow-up in months	14.2 (8-38)	0.3-211.5
Transplant toxicity		
A. Infectious complications	Number	Percentage (%)
*Staphylococcus aureus*	8	8.7
*Acinetobacter baumani*	5	5.4
*Enterococcus faecium*	5	5.4
*Escherichia coli*	2	2.2
*Clostridium difficile*	2	2.2
*Giardia lamblia*	2	2.2
*Candida* sp.	2	2.2
B. Non-infectious complications (≥Grade 3)		
Mucositis	38	41.3
Chemotherapy-induced nausea vomiting	21	22.8
Diarrhea	28	30.4
Engraftment syndrome	7	7.6
Acute kidney injury	4	4.3
Transplant-related mortality (TRM)	4	4.3

ASCT, autologous stem cell transplantation; ANC, absolute neutrophil count; PRBC, packed red blood cells; SDP, single donor platelets; CR, complete remission; No CR, not in complete remission; CD34, cluster of differentiation 34; IQR, interquartile range; TRM, transplant-related mortality.

*Includes two patients each of rhabdomyosarcoma (RMS) and non-rhabdomyosarcoma soft-tissue sarcomas (NRSTS).

#Two patients were in stable disease (SD), while the rest were in partial response (PR).

### Transplant details

The median dose of CD34+ stemcells infused was 5.7 × 10^6^/kg (IQR, 3.5-6.9 × 10^6^/kg) with the median duration of cryopreservation being 16 days (IQR, 8-49). Neutrophil engraftment occurred at a median of 11 days (IQR, 9-12). Median time for platelet engraftment was not available as most of the patients were discharged before platelet engraftment and received platelet transfusions on daycare basis in view of limitation of beds. The median number of packed red blood cell (PRBC) transfusions required was 1 (IQR, 1-2), and the median number of single-donor platelet (SDP) transfusions during hospitalization was 2 (IQR,1-4.5). Patients received a median of 10 days (IQR, 7-13) of intravenous antibiotics, with the median duration of hospitalization for ASCT being 23 days (IQR, 20-28).

### Outcomes and complications

**Figure 1:**
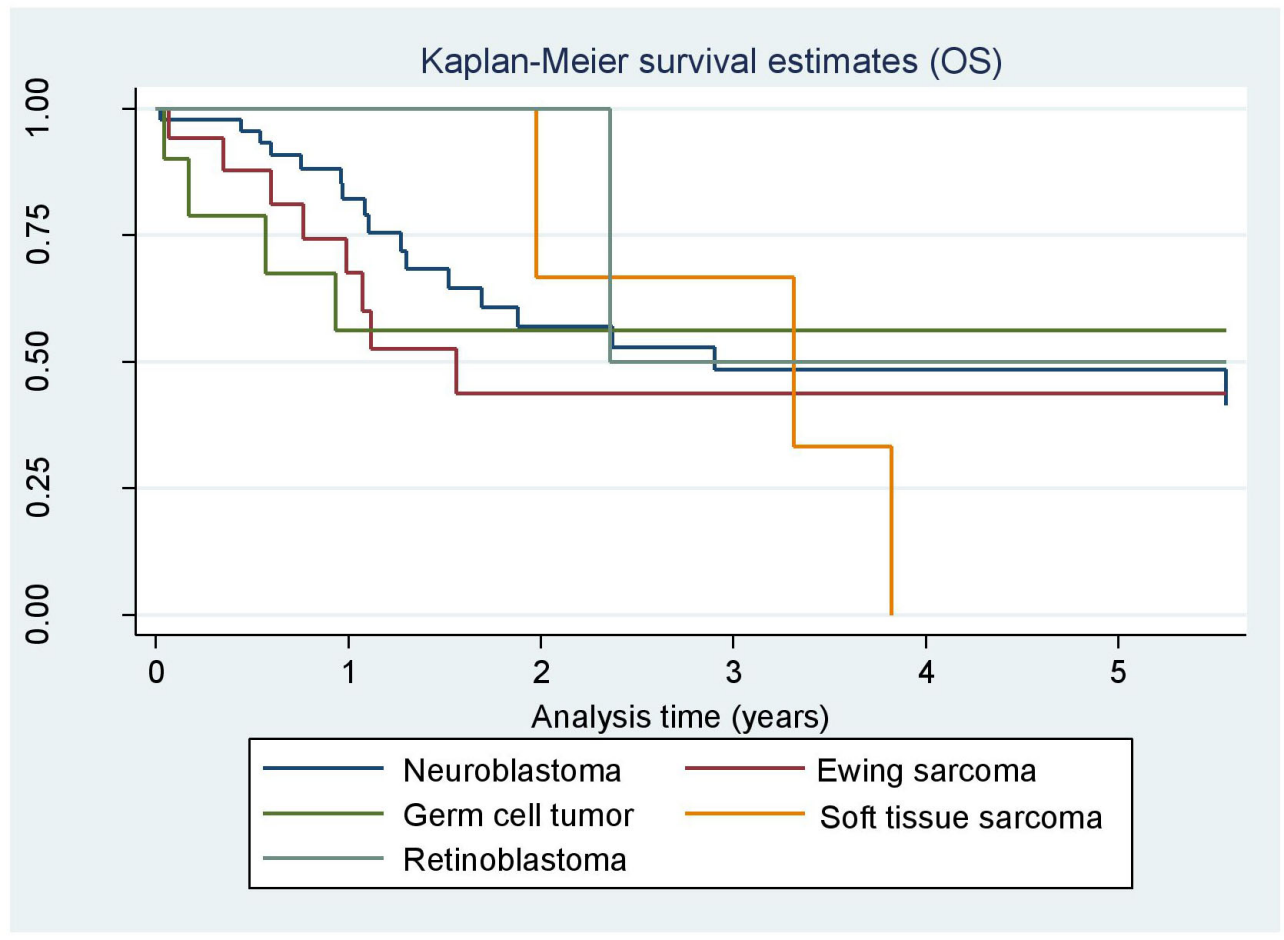
Kaplan Meier survival curve for overall survival (OS) based on diagnosis.

**Figure 2:**
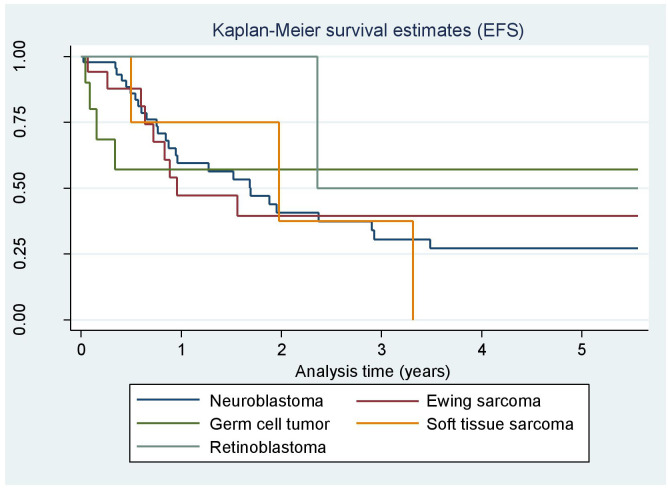
Kaplan Meier survival curve for event-free survival (EFS) based on diagnosis.

The median duration of post-transplant follow-up was 14.2 months (IQR, 8-38). The 3-year OS of the entire cohort was 50.6% (95%CI: 36.8-62.9), and EFS was 35.9% (95%CI: 23.6-48.3). The disease wise 3-year OS for HRNB, relapsed/refractory EWS, relapsed/refractory GCT, STS and RB were 48.5%(95%CI: 29.2-65.3), 43.8% (95%CI: 17.8-67.4), 56.3%(95%CI: 20.9-80.9), 66.7% (95%CI: 5.4-94.5), and 50.0%(95%CI: 6.0-91.0) respectively. The corresponding 3-year EFS were 30.6% (95% CI: 16.0–46.5), 39.4% (95% CI: 12.9–62.7), 56.3%(95%CI: 20.9-80.9), 37.5% (95% CI: 28.6–80.8), and 50.0% (95% CI: 0.6–91.0), respectively (**Figures 1**, **2**). Details of the infectious and non-infectious complications are mentioned in **Table 1**. Non-infectious toxicities of grade ≥3 severity included mucositis in 38(41.3%) patients, chemotherapy-induced nausea and vomiting in 21(22.8%), and diarrhea in 28(30.4%). Acute kidney injury of greater than grade 3 was reported in 4 patients, all of whom were relapsed GCTs with high cumulative doses of cisplatin. There were three (3.2%) transplant-related mortalities (TRM) within 30 days post-ASCT and four (4.3%) TRM by day 100. Causes of death included sepsis with multi-organ dysfunction syndrome in three patients and acute kidney injury (AKI) in one.

### Factors predicting survival

On univariate analysis (**Table 2**), female sex was significantly associated with improved OS (HR: 0.34; 95% CI: 0.14-0.84; p=0.009) and EFS (HR: 0.42; 95% CI: 0.20-0.88; p=0.022) compared to males. Diagnosis and age at transplant did not have a statistically significant impact on OS or EFS. Patients undergoing ASCT in the relapse/refractory setting had inferior OS (HR: 3.33; 95% CI: 1.01-10.91; p=0.048) compared to those receiving it upfront, with a similar trend for EFS (HR: 2.11; 95% CI: 0.89-4.95; p=0.086); however, these cohorts represent biologically and clinically distinct disease entities and may not be directly comparable. Pre-ASCT disease status also showed a trend toward worse EFS in patients not in complete remission versus those in CR (HR: 2.22; 95% CI: 0.90–5.49; p=0.072), although this did not reach statistical significance for OS. Along with sex, the latter two variables were included in multivariate analysis due to a trend toward significance. On multivariate analysis (**Table 3**), female sex remained a significant protective predictor of both OS (HR: 0.11; 95% CI: 0.02–0.84; p=0.034) and EFS (HR: 0.18; 95% CI: 0.05–0.62; p=0.007). ASCT performed in the relapse/refractory setting independently predicted inferior OS (HR: 5.62; 95% CI: 1.59–19.86; p=0.007) and EFS (HR: 3.72; 95% CI: 1.47–9.38; p=0.005). Pre-transplant disease status was also an independent predictor of worse outcomes, with significantly inferior EFS (HR: 3.22; 95% CI: 1.22–8.46; p=0.018) and OS (HR: 3.50; 95% CI: 1.01–12.23; p=0.049) in patients not in remission.

**Table 2:** Univariate analysis of factors predicting OS and EFS.

Variables	Overall survival			Event free survival		
	HR	[95% Conf interval]	p-value	HR	[95% Conf interval]	p-value
**Age**	1.02	0.97-1.06	0.349	1.02	0.98- 1.06	0.389
Sex						
Male	Ref					
Female	0.34	0.14-0.84	**0.009**	0.42	0.20-0.88	**0.022**
Diagnosis						
Neuroblastoma	Ref			Ref		
Ewing sarcoma	1.39	0.59-3.22	0.444	0.99	0.46-2.11	0.975
Germ cell tumor	1.05	0.35-3.12	0.937	0.83	0.29-2.37	0.723
Soft tissue sarcoma	1.28	0.37-4.39	0.697	1.11	0.34-3.68	0.862
Retinoblastoma	0.68	0.09-5.15	0.711	0.43	0.06-3.19	0.410
Indication of transplant						
First remission	Ref					
Relapse/refractory	3.33	1.01-10.91	**0.048**	2.11	0.89-4.95	**0.086**
Pre-ASCT status						
CR	Ref					
Not in CR	2.38	0.72-7.79	0.136	2.22	0.90-5.49	**0.072**
Conditioning regimen (n-62)						
CEM-200	Ref					
BuMel-140	0.15	0.04-0.70	**0.005**	0.60	0.23-1.57	0.302

HR, hazard ratio; CI, confidence interval; ASCT, autologous stem cell transplantation; CR, complete remission; No CR, not in complete remission; CEM, carboplatin–etoposide–melphalan; BuMel, busulfan–melphalan.

Bold values indicate p<0.1.

**Table 3:** Multivariate analysis of factors predicting OS and EFS.

Variables	Overall survival			Event-free survival		
	HR	[95% Conf interval]	p-value	HR	[95% Conf interval]	p-value
Sex						
Male	Ref					
Female	0.11	0.02-0.84	**0.034**	0.18	0.05-0.62	**0.007**
Indication of transplant						
First remission	Ref					
Relapse/refractory	5.62	1.59-19.86	**0.007**	3.72	1.47-9.38	**0.005**
Pre-ASCT status						
CR	Ref					
Not in CR	3.50	1.01-12.23	**0.049**	3.22	1.22-8.46	**0.018**

HR, hazard ratio; CI, confidence interval; ASCT, autologous stem cell transplantation; CR, complete remission; No CR, not in complete remission.

Bold values indicate p<0.05.

The conditioning regimen (BuMel-140 or CEM-200) was analyzed solely in the univariate model, as its relevance was limited to patients with HRNB and relapsed/refractory EWS. Among these regimens, BuMel-140 conditioning was associated with significantly superior OS compared to CEM-200 on univariate analysis (HR: 0.15; 95% CI: 0.04–0.70; p=0.005), although there was no statistically significant difference in EFS (HR: 0.60; 95%CI:0.23-1.57; p=0.302).

## Discussion

This 15-year single-center, high-volume ASCT experience demonstrates that it can achieve acceptable and clinically meaningful outcomes as a consolidation strategy for selected high-risk or relapses/refractory solid tumors in CAYA, despite the limited supportive care infrastructure in resource-constrained settings. ASCT in these cases was delivered along with the multimodality treatment, comprising systemic chemotherapy with appropriate surgical and radiotherapeutic local and metastatic disease control. Beyond neuroblastoma, the study also reports outcomes for other relatively uncommon pediatric solid tumors in which ASCT remains a therapeutic option. Notably, it included rare indications such as retinoblastoma and soft-tissue sarcomas, for which published evidence from LMICs is largely lacking (8, 9). These outcomes were achieved despite the unavailability of busulfan pharmacokinetic monitoring and HEPA-filtration facilities, while maintaining a low day+100 transplant-related mortality of 4.3%, comparable to other regional reports in high-risk neuroblastoma (10, 11). In addition, the availability of plerixafor allowed successful stem cell mobilization in heavily pretreated pediatric solid tumor patients, resulting in adequate CD34+ cell yields (12).

Although the outcomes of neuroblastoma in our cohort are inferior to contemporary results achieved with dinutuximab-based immunotherapy (13), they exceed the 0–35% OS reported for HRNB treated without ASCT in LMICs (14, 15) and are comparable to outcomes from the pre-dinutuximab era in HICs (16). Early reports suggest that some HRNB patients may achieve adequate outcomes with anti-GD2 therapy even without ASCT (17). However, a recent systematic review suggests that, given the poor outcomes following relapse and the lack of randomized evidence supporting omission of ASCT, it remains an integral part of HRNB therapy until further studies identify patient groups that can safely receive earlier anti-GD2 immunotherapy without ASCT (18). In this context, our findings reinforce the continued relevance of ASCT in HRNB within LMIC settings, where access to anti-GD2 immunotherapy remains limited.

Similarly, survival for relapsed/refractory EWS in our cohort was 43.8%, exceeding the <15% survival reported in non-ASCT cohorts from other settings (19). However, this remains lower than outcomes reported in Western series (50–60%), likely due to the use of less intense salvage regimens, given the challenges in delivering dose-intensive approaches such as high-dose ifosphamide (19, 20). In the absence of randomized evidence, four small retrospective studies summarized by Ramamurthy et al. have demonstrated better outcomes with ASCT compared to conventional chemotherapy in relapsed/refractory EWS (20). For other relapsed soft-tissue sarcomas, the small number of cases in our cohort limits definitive conclusions, although some carefully selected good responders achieved encouraging survival. Globally, no prospective randomized studies evaluating ASCT in relapsed/refractory soft-tissue sarcoma are available; existing evidence is largely derived from retrospective analyses of American/European registries. Most reported ASCTs in rhabdomyosarcoma (RMS) have been performed in high-risk and/or metastatic disease, with minimal data addressing the relapsed/refractory setting separately (20). For non-rhabdomyosarcoma soft-tissue sarcomas (NRSTS), a Cochrane review highlighted no survival advantage for ASCT based on a single randomized trial with an unclear risk of bias and moderate to high quality evidence (21). Against this background, our data contribute to the limited literature on ASCT for relapsed/refractory Ewing sarcoma and other soft-tissue sarcomas in LMICs.

In contrast to HRNB and sarcomas, for relapsed GCT, the 3-year OS of 56.3% in our study is similar to the outcomes from HICs and slightly favorable compared to other LMIC reports, where an OS of 47.1% was observed following ASCT with carboplatin–etoposide–melphalan conditioning (22). While the ongoing TIGER trial will give the most definitive evidence on whether ASCT is better than conventional chemotherapy alone in relapsed GCT, our data reinforce that even in LMIC settings, outcomes of relapsed GCT can reach the global standards, after platinum-based salvage followed by ASCT (3).

For retinoblastoma, although the ARET 0321 trial evaluated upfront consolidative ASCT in advanced disease and demonstrated encouraging short-term survival in selected subgroups, evidence supporting ASCT in metastatic or relapsed/refractory retinoblastoma remains largely limited to small case series, with sparse real-world data from LMIC settings (23). In our study, only five patients with retinoblastoma underwent ASCT, and although the observed survival was 50%, the small number precludes any definitive conclusions. However, the experience suggests that the procedure delivers acceptable outcomes in patients with chemosensitive disease.

The finding of a significant survival advantage observed in females over males in both univariate and multivariate analysis in this study likely reflects the gender differences in outcomes of solid tumors, rather than factors specific to ASCT. Similar findings of better outcomes in females were reported in a study that included 10,534 cases of extracranial solid tumors of childhood from the Surveillance, Epidemiology, and End Results (SEER) database diagnosed between 1985 and 2005 (24). Although the precise mechanism of this difference is not clear, it probably reflects a combination of factors, including the tumor biology in females, better response, sex-based differences in pharmacogenetics or drug metabolism, and possible hormonal influences on immune functions (25, 26). In line with the existing literature, inferior survival was reported in patients transplanted in relapse/refractory settings as well as in those without complete remission, underscoring the importance of optimal disease control before ASCT (27).

A separate univariate analysis in neuroblastoma and Ewing sarcoma patients revealed a significantly better OS with the BuMel-140 regimen over CEM-200. This is supported by the findings from the HR-NBL1/SIOPEN trial, which established the superiority of BuMel-140 in terms of OS, EFS, as well as toxicity, over the CEM regimen that included melphalan 200mg/m^2^ along with carboplatin and etoposide (28). In our relatively smaller cohort, the difference in EFS did not reach statistical significance, which may be attributed to the fact that the observed benefit was primarily due to a reduction in treatment-related mortality rather than a decrease in relapse incidence. Infection was the principal cause of TRM in our cohort, with several patients experiencing microbiologically documented bacterial infections; however, the overall TRM remained low and comparable to global standards (29). Three patients with relapsed GCT developed acute kidney injury and later progressed to chronic kidney disease; all had received cumulative cisplatin doses exceeding 600 mg/m² during primary and salvage therapy (30). It suggests that in patients with high cumulative cisplatin exposure (≥400–500mg/m²), further platinum use during salvage therapy should be judicious, and in cases where this threshold has already been exceeded, ASCT conditioning should favor non-cisplatin regimens, such as melphalan-based conditioning (31).

This study represents comprehensive evidence for the efficacy of ASCT in a variety of pediatric solid tumors, despite the limited resources in LMICs. This also underscores the unmet need for life-saving immunotherapies, like anti-GD2 antibodies, for otherwise fatal diseases like HRNB. The limitations of the study include the heterogeneous study population, small subgroups for certain tumor types, and the long study period, during which supportive care would have improved, contributing partly to the differences in outcomes between the conditioning regimens. We also acknowledge the limited representation of brain tumors in our cohort, as the feasibility of tandem ASCT is limited in LMICs like ours. Non-availability of busulfan pharmacokinetics and HEPA filtration facility was also another limitation. Although a formal cost analysis was not conducted, the delivery of ASCT without these high-cost supportive care infrastructures likely reduced the overall cost in our setting (~3500 USD) in comparison to HICs (~100,000 USD) (32). This cost structure reflects delivery within a government-funded public sector tertiary care hospital in India and may not be generalizable to private sector health-care systems. With government and philanthropic support, such lower-cost delivery models enable ASCT to achieve acceptable outcomes in LMICs at substantially lower cost, although with some outcome lag behind the HICs with better supportive care and novel agents. Emerging immunotherapeutic approaches, including chimeric antigen receptor T-cell (CAR-T) therapy targeting GD2 and other tumor-associated antigens, are also being explored in pediatric solid tumors, particularly in relapsed/refractory disease and in patients unsuitable for ASCT. However, these strategies remain investigational, with limited long-term efficacy data and restricted accessibility in most LMIC settings (33).

To conclude, ASCT results in acceptable survival outcomes for selected high-risk and relapsed/refractory pediatric solid tumors in LMICs. While for relapsed/refractory GCT, the outcomes are comparable to HICs, for diseases like HRNB and Ewing sarcoma, parallel efforts to improve access to immunotherapy and better supportive care are needed. Broader availability of transplant facilities, public-private partnership, optimal pre-ASCT disease control, and integration of emerging therapies may further enhance the survival and match the outcomes to global standards.

## Data Availability

The raw data supporting the conclusions of this article will be made available by the authors, without undue reservation.
