# Supplementary material for: Autologous stem cell transplantation for pediatric solid tumors in a resource-limited setting: a single-center experience of 15 years
Source: Front Oncol. 2026 Jun 16;16:1796956. doi: 10.3389/fonc.2026.1796956 (PMC13314500; doi:10.3389/fonc.2026.1796956)
Supplement: Supplementary file 1 [file Table1.docx]

**Supplementary Table 1: Conditioning regimens used as per individual tumor subtypes**

| **Tumor Type** | **Conditioning Regimen (Drug, Dose, and Days)** |
| --- | --- |
| **Neuroblastoma and Ewing sarcoma** | **CEM-200** (Mel-200): *(used till 2011)*   - Carboplatin: 375 mg/m²/day IV on Days -7, -6, -5, and -4 - Etoposide: 300 mg/m²/day IV on Days -7, -6, -5, and -4 - Melphalan: 100 mg/m²/day IV on Days -6 and -5   **BuMel-140**: *(post-2011)*   - Busulfan: 16 mg/kg IV (divided as 4 mg/kg/day × 4 days; Day -5 to -2) - Melphalan: 140 mg/m² IV on Day -1 |
| **Germ Cell Tumors (GCT)** | - Carboplatin: AUC 5–7 (approx. 500–700 mg/m²/day) IV on Days -5 to -3 - Etoposide: 400 mg/m²/day IV on Days -5 to -3 |
| **Retinoblastoma and soft tissue sarcoma*** | - Carboplatin: 500 mg/m²/day IV on Days -8 to -6 - Etoposide: 400 mg/m²/day IV on Days -5 to -3 |

* Thiotepa at a dose of 300 mg/m²/day IV on Days -4 and -3 was added in three patients with retinoblastoma and two with soft tissue sarcoma
